# Biliary Intraepithelial Neoplasia With Gallbladder Adenoma and Cirrhosis: A Case Report

**DOI:** 10.7759/cureus.27780

**Published:** 2022-08-08

**Authors:** William J Chen, Evan Wolff, Chintalapati R Varma, Ramy Shoela

**Affiliations:** 1 Radiology, Saint Louis University School of Medicine, St. Louis, USA; 2 Surgery, Saint Louis University Hospital, St. Louis, USA

**Keywords:** cystic duct, common bile duct, intraductal papillary neoplasm of bile duct, liver transplant, cirrhosis, cholangiocarcinoma, biliary intraepithelial neoplasia, gallbladder adenoma

## Abstract

Biliary intraepithelial neoplasia (BilIN) is a precursor lesion of cholangiocarcinoma that has been rarely reported. The present study reports a 56-year-old male with low-grade BilIN of the bile ducts and the cystic duct margin. Stent exchange endoscopy demonstrated an irregular, intraductal mass extending along the common bile duct, common hepatic duct, and hepatic duct bifurcation. The peribiliary mass was found to abut the right portal vein, inferior vena cava, and pancreatic head, and replaced the right hepatic artery. In addition, there was evidence of gallbladder adenoma managed with cholecystectomy and a right-lobed liver lesion and cirrhosis, which prompted the discussion of prophylactic liver transplantation. We emphasize the radiological features of BilIN and associated pathological findings through multiple imaging modalities. Consideration of this diagnosis is indicated in western countries and requires timely management based on available guidelines.

## Introduction

Biliary intraepithelial neoplasia (BilIN) is characterized as a spectrum of proliferative, flat, or micropapillary precursor lesions of bile duct adenocarcinoma [1]. BilIN ranges from low-grade (I), intermediate-grade (II), and high-grade BilIN (III), representing carcinoma in situ. BilIN is considered to be the primary pathway that progresses to intrahepatic cholangiocarcinoma through a dysplasia-carcinoma sequence [2]. While the incidence of the disease is not well established, one study has demonstrated that 29.5% of patients with cholangiocarcinoma were found to have BilIN, indicating its prevalence among cholangiocarcinoma patients [3].

Based on the 2010 World Health Organization classification, intraductal papillary neoplasm of the bile duct (IPNB) is defined as a papillary or villous neoplasm covering the fibrovascular stalks occurring in the bile ducts. Imaging features of IPNB vary according to the presence of intraductal lesions, degree of mucin production, and tumor location [4]. While BilIN is a flat or low-papillary microscopic lesion that does not produce grossly or radiologically detectable mass, IPNB is a macroscopic lesion that produces grossly and radiologically detectable mass lesion [1]. Both BilIN and IPNB are considered precursor lesions of cholangiocarcinoma with higher prevalence in eastern countries like Korea, Japan, and China [4].

Due to the poor prognosis of cholangiocarcinoma, it is critical to identify associated risk factors. In western countries, the major risk factors for biliary neoplasia are primary sclerosing cholangitis, Thorotrast deposition, abnormal choledochopancreatic junction, and choledochal cysts. In Southeast Asian countries, intrahepatic cholangiocarcinoma is more frequent than extrahepatic cholangiocarcinoma with predisposing factors of hepatolithiasis and parasitic infection [5]. Additional risk factors include chronic hepatitis C and alcoholic cirrhosis, which are associated with small intrahepatic bile duct neoplasia.

A majority of reported BilIN presents as lesions that do not form a mass or cause bile duct obstruction. However, when these tumors grow large enough, symptoms of biliary obstruction such as jaundice, intermittent pain, dyspepsia, weight loss, nausea, and vomiting become present [6]. To our knowledge, the presenting case features low-grade IPNB lesions with gallbladder adenoma and cirrhosis that have not been previously described.

## Case presentation

A 56-year-old male with no prior medical history and no history of smoking or heavy alcohol use presented with itching, very sporadic postprandial bloating, and light-colored stools. His most recent colonoscopy indicated no polyps or concerning lesions, but the presence of diverticulosis was noted. He denied changes to stool caliber, melena, hematochezia, fever, chills, night sweats, or any unplanned weight loss. Labs were significant for elevated total bilirubin, elevated alkaline phosphate, and abnormal liver function tests. The patient was negative for hepatitis A, B, and C, though there was an abnormal Epstein-Barr virus (EBV) result.

**Figure 1:**
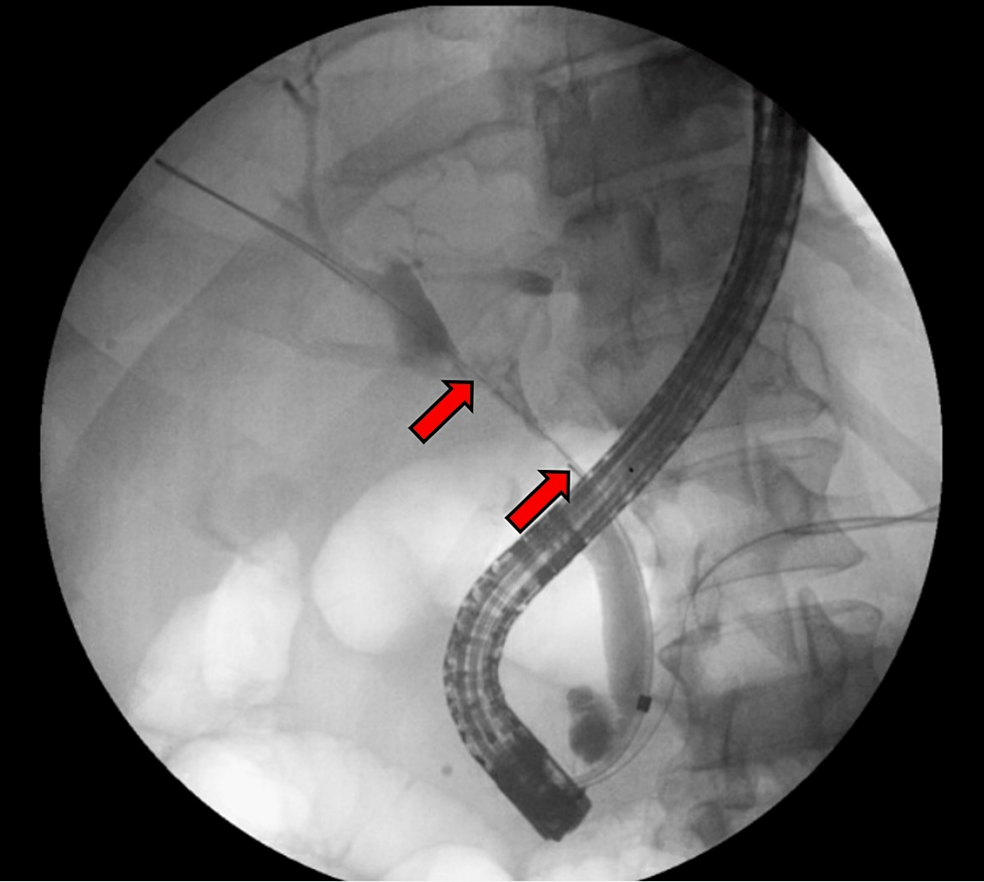
Endoscopic retrograde cholangiopancreatography demonstrates bile duct obstruction Endoscopic retrograde cholangiopancreatography indicates bile duct obstruction from an intraductal soft tissue lesion. Contrast injection above the level of filling defect showed a markedly dilated left and right hepatic duct, estimated to be approximately 10 to 12 mm on each side. The filling defect was estimated to be approximately 20 mm in length (red arrows). There was some notable filling defect within the left intrahepatic duct as well.

**Figure 2:**
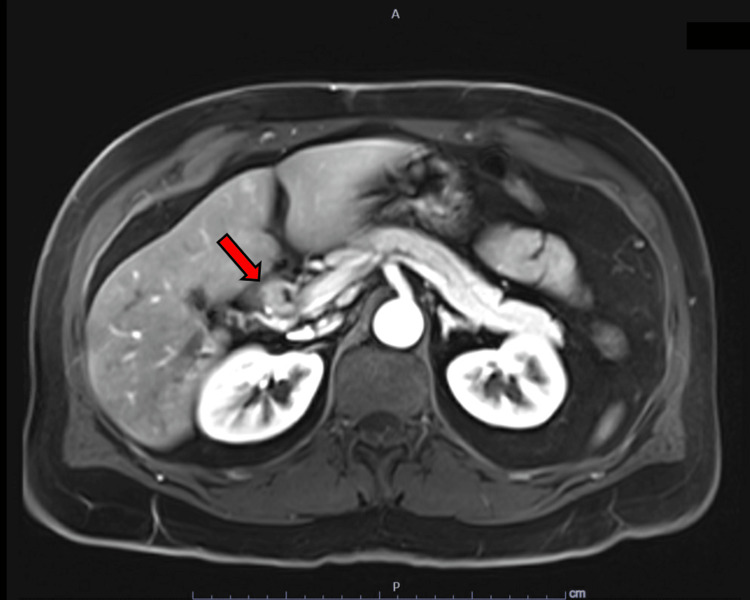
MRI arterial post-contrast demonstrates biliary mass Soft tissue (red arrow) extends over a length of greater than 4.2 cm and measures up to 1.8 cm in width. There is an additional enhancing soft tissue within the common bile duct at the bifurcation and extends to the right intrahepatic bile duct.

**Figure 3:**
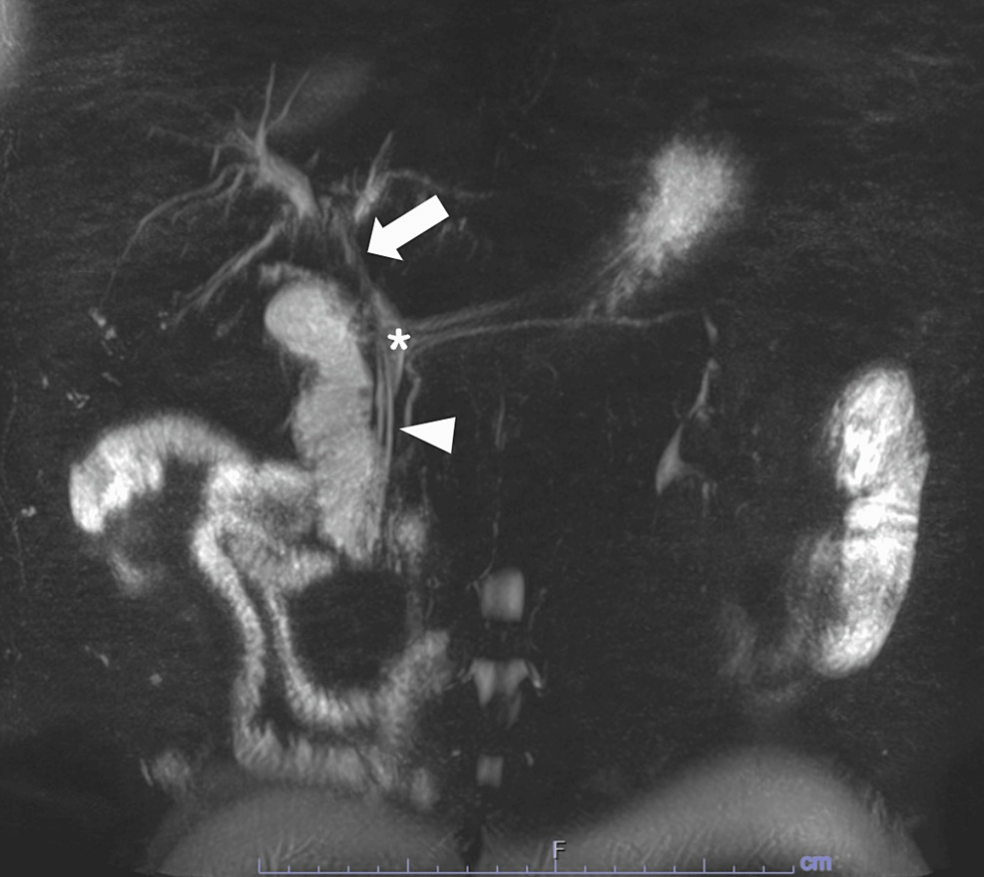
Magnetic resonance cholangiopancreatography with maximum intensity projection There is mild intrahepatic and extrahepatic bile duct dilatation. There is a long segment 2.9 cm stricture of the hilar common hepatic duct (arrows). A linear filling defect within the common bile duct is consistent with a known biliary stent (arrowhead). Peribiliary mass (asterisk) measuring up to 1.5 x 1.2 cm is unchanged, abutting the right portal vein, inferior vena cava, and pancreatic head, and replacing the right hepatic artery. Postcontrast enhancement is noted throughout the common bile duct.

**Figure 4:**
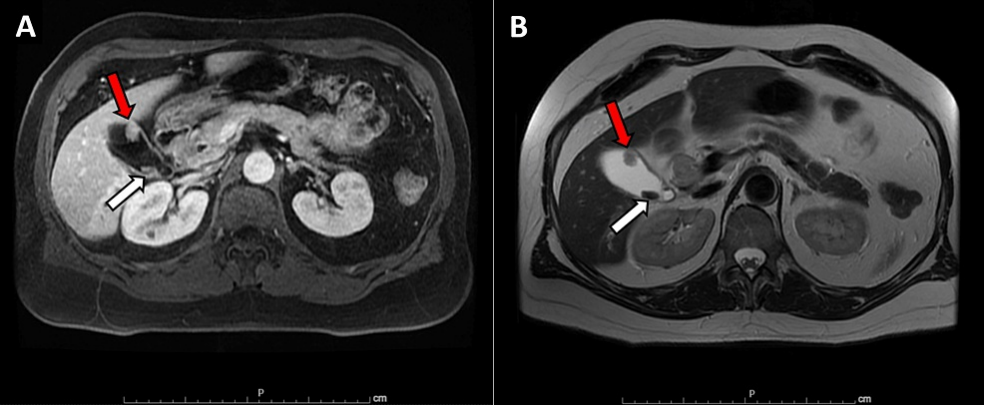
MRI showing gallbladder adenoma and intraluminal gallstone (A) MRI venous phase post-contrast shows a 1.6 x 1.2 cm enhancing mass in the gallbladder (red arrow) and intraluminal gallstone (white arrow). (B) T2 half-Fourier acquisition single-shot turbo spin-echo (HASTE) shows the same findings as above.

**Figure 5:**
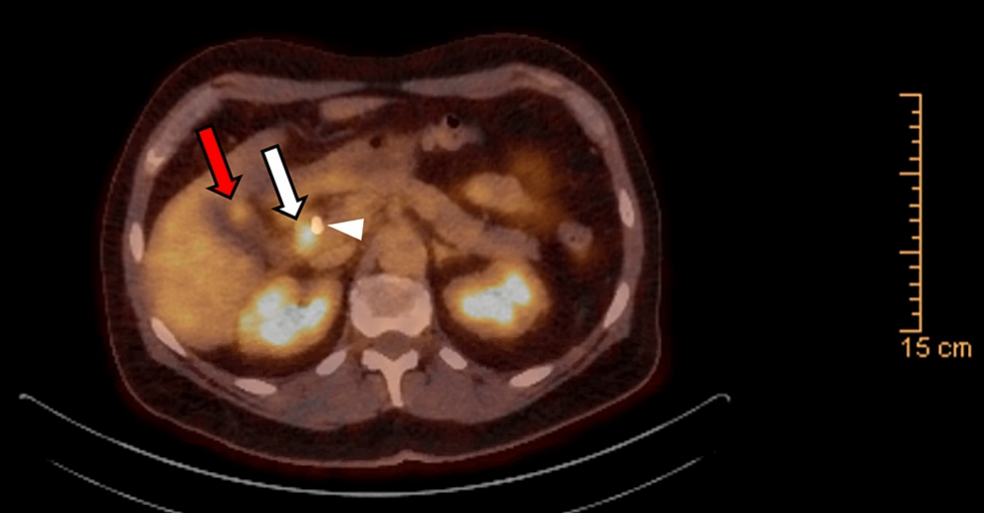
Positron emission tomography demonstrates gallbladder and biliary malignancies There is a 0.9 x 1.4 cm hyperdense lesion in the nondependent portion of the gallbladder (red arrow) with a standardized uptake value (SUV) max of 4.4, suspicious for malignancy. In addition, there is intense fluorodeoxyglucose (FDG) uptake along the biliary stent (arrowhead), SUV max of 7.3, likely representing biliary malignancy (white arrow).

He was referred for an abdominal ultrasound, which showed dilation of the common bile duct. He then underwent endoscopic ultrasound, endoscopic retrograde cholangiopancreatography, and biopsy of proximal common bile duct intraductal stricturing lesion along with stent placement (Bismuth IV biliary stricture extending from the common bile duct to bilateral intrahepatic ducts; Figure 1). Combined ultrasound and MRI showed concerning lesions in the gallbladder, right intrahepatic duct, and proximal common bile duct that was biopsied. The mass in the bile duct abuts the pancreatic head, inferior vena cava minimally, and duodenum. It replaced the right hepatic artery (Figure 2) and caused stenosis of the common bile duct (Figure 3). A 1.3-cm soft tissue nodule was also detected in the gallbladder in addition to a gallstone (Figure 4). Cholecystectomy was performed and pathology indicated the gallbladder nodule to be an intracholecystic papillary neoplasm (ICPN) with low-grade dysplasia. In addition, biopsies revealed a low-grade IPNB (BillN-1/2) and a focal low-grade BilIN present at the cystic duct margin (BillN-1). Liver lesion biopsy showed focal bridging fibrosis and nodularity. Positron emission tomography (PET) scan demonstrated intense fluorodeoxyglucose (FDG) uptake along the biliary stent and FDG-avid lesion within the gallbladder (Figure 5). Cholecystectomy was performed based on these findings.

On stent exchange endoscopy, there was an irregular isoechoic intraductal mass extending along the common bile duct, common hepatic duct, and hepatic duct bifurcation. Due to extensive intraductal papillary mucinous neoplasm, the patient was discussed at the liver selection meeting and deemed a good candidate with conventional hepatic anatomy. The patient underwent an orthotopic liver transplant and a Whipple's procedure with a two-week interval between both procedures. Pathological evaluation of the explanted native liver demonstrated high-grade IPNB with a focal invasive carcinoma (1-2 mm) at the confluence of the right and left hepatic ducts. The subsequent Whipple's procedure showed focal high-grade intraductal papillary neoplasm of the distal common bile duct to the proximal margin to the ampulla. There was no evidence of malignancy in the pancreatic head, duodenum, distal stomach, or lymph nodes.

## Discussion

The association between BilIN, gallbladder adenoma, and cirrhosis is not well understood. Comparison of high-grade BilIN with ICPN of gallbladder suggests that conglomerated ICPNs may arise from high-grade BilINs with short papillary components [7]. A prior study reports two cases of biliary neoplasia with extensive intraductal spread arising in liver cirrhosis, suggesting the possibility that biliary neoplasia with intraductal spread may be a variant of BilIN [8]. In a study investigating the prevalence of BilIN in non-biliary causes of cirrhosis, 31% of cases (n = 100) demonstrated BilIN-1, 2% of cases showed BilIN-2, and no cases of BilIN-3 were reported, which suggests that non-biliary causes of cirrhosis should be considered as precursors of cholangiocarcinoma [9]. There is evidence indicating that chronic hepatitis C virus and alcohol cirrhosis are risk factors for intrahepatic cholangiocarcinoma. However, our patient had neither risk factors, which may suggest other possible risk factors. Interestingly, EBV may cause liver injury and even liver failure, with the association with autoimmune liver diseases including autoimmune hepatitis, primary biliary cholangitis, and primary sclerosing cholangitis [10-13]. Although our patient’s abnormal EBV antibody IgG may suggest an infectious etiology resulting in acute liver injury, there has not been any study of EBV infection predisposing patients to cirrhosis.

Due to the poor prognosis of gallbladder cancer, every attempt must be made to identify asymptomatic stages and premalignant gallbladder polyps and adenoma. Gallbladder polyps larger than 1.5 cm, especially in solitary sessile hypoechogenic polyps, are associated with a risk of malignancy and require cholecystectomy when there are symptoms such as biliary-type pain and dyspepsia [14]. Indications for resection in asymptomatic patients include age greater than 50 years old, polyp size greater than 10 mm, concurrent gallstone, or continuous polyp growth on ultrasound exams [15]. Gallbladder adenoma is a rare benign epithelial tumor that typically presents as a pedunculated solitary lesion with sizes ranging from 5 to 20 mm and is categorized as pyloric, intestinal, foveolar, or biliary [16]. The 2019 WHO classification of tumors of the digestive system proposed ICPN as a preinvasive neoplasm of the gallbladder that presents as an intraluminal mass lesion [17]. The prognosis for ICPN is better than that for invasive gallbladder carcinomas, with three-year survival averaging 90% for lesions with no foci of invasion versus 60% for those with foci of invasion [18]. The presence of low-grade dysplasia of ICPN with no evidence of invasive foci has previously been managed without intervention or additional testing [19]. However, our patient met the indications for resection and therefore underwent cholecystectomy similar to prior reported cases [15].

Prior report of BilIN-2 in the common bile duct has been successfully treated with local excision with no evidence of recurrence during a three-year follow-up period; however, this treatment strategy may be limited to managing the less common extrahepatic BilIN [6]. Another case report features variable BilIN ranging from low to high grade in a patient with benign biliary stricture with imaging modalities revealing dilation of left intrahepatic bile ducts with focal narrowing of the left hepatic duct. Due to the risk of possible cholangiocarcinoma, the patient underwent left liver lobectomy [20]. Given the unique findings of our case report with the presence of BilIN, cirrhosis, and gallbladder adenoma, our therapeutic approach in addition to the cholecystectomy is to perform a prophylactic liver transplant with the concern of malignant transformation.

## Conclusions

BilIN is a rare disease with few instances reported. This case study features a unique presentation of BilIN with imaging modalities highlighting an extrahepatic biliary mass. Understanding the natural history of the disease with possible progression to cholangiocarcinoma is valuable due to the poor prognosis associated with cholangiocarcinoma. The case provides insight into a rare disease with complex features, including low-grade IPNB with gallbladder adenoma and cirrhosis. Though the prevalence is low for a pathologic diagnosis of BilIN, there is still a need for further investigation to provide a greater understanding of disease progression and management.

## References

[1] Zen Y, Adsay NV, Bardadin K (2007). Biliary intraepithelial neoplasia: an international interobserver agreement study and proposal for diagnostic criteria. Mod Pathol.

[2] Wu TT, Levy M, Correa AM, Rosen CB, Abraham SC (2009). Biliary intraepithelial neoplasia in patients without chronic biliary disease: analysis of liver explants with alcoholic cirrhosis, hepatitis C infection, and noncirrhotic liver diseases. Cancer.

[3] Yoon KC, Yu YD, Kang WH, Jo HS, Kim DS, Kim JY (2019). Prevalence and clinical significance of biliary intraepithelial neoplasia (BilIN) in cholangiocarcinoma. Am Surg.

[4] Park HJ, Kim SY, Kim HJ (2018). Intraductal papillary neoplasm of the bile duct: clinical, imaging, and pathologic features. AJR Am J Roentgenol.

[5] Ainechi S, Lee H (2016). Updates on precancerous lesions of the biliary tract: biliary precancerous lesion. Arch Pathol Lab Med.

[6] Wang W, Chen W, Li K, Wang J (2016). Successful treatment of biliary intraepithelial neoplasia in the common bile duct via local excision: a case report. Oncol Lett.

[7] Nakanuma Y, Sugino T, Okamura Y, Nomura Y, Watanabe H, Terada T, Sato Y (2021). Characterization of high-grade biliary intraepithelial neoplasm of the gallbladder in comparison with intracholecystic papillary neoplasm. Hum Pathol.

[8] Aishima S, Nishihara Y, Tsujita E (2008). Biliary neoplasia with extensive intraductal spread associated with liver cirrhosis: a hitherto unreported variant of biliary intraepithelial neoplasia. Hum Pathol.

[9] Zarei M, Shasaeefar A, Kazemi K, Dehghani M, Malekhosseini SA, Geramizadeh B (2019). Biliary intraepithelial neoplasia in non-biliary cirrhosis—report from 100 explanted livers: a single center experience. Clin Pathol.

[10] Mellinger JL, Rossaro L, Naugler WE, Nadig SN, Appelman H, Lee WM, Fontana RJ (2014). Epstein-Barr virus (EBV) related acute liver failure: a case series from the US Acute Liver Failure Study Group. Dig Dis Sci.

[11] Zhang W, Chen B, Chen Y (2016). Epstein-Barr virus-associated acute liver failure present in a 67-year-old immunocompetent female. Gastroenterology Res.

[12] Gupta E, Ballani N, Kumar M, Sarin SK (2015). Role of non-hepatotropic viruses in acute sporadic viral hepatitis and acute-on-chronic liver failure in adults. Indian J Gastroenterol.

[13] Rigopoulou EI, Smyk DS, Matthews CE, Billinis C, Burroughs AK, Lenzi M, Bogdanos DP (2012). Epstein-Barr virus as a trigger of autoimmune liver diseases. Adv Virol.

[14] Matos AS, Baptista HN, Pinheiro C, Martinho F (2010). Gallbladder polyps: how should they be treated and when?. Rev Assoc Med Bras (1992).

[15] Yang G, Qin H, Raza A, Saukel GW, Solomon N, Michelotti M, Raghavan R (2016). Pyloric gland adenoma of gallbladder—reports of two cases and a brief review of literature. J Gastrointest Oncol.

[16] Andrén-Sandberg A (2012). Diagnosis and management of gallbladder polyps. N Am J Med Sci.

[17] Nagtegaal ID, Odze RD, Klimstra D (2020). The 2019 WHO classification of tumours of the digestive system. Histopathology.

[18] Adsay V, Jang KT, Roa JC (2012). Intracholecystic papillary-tubular neoplasms (ICPN) of the gallbladder (neoplastic polyps, adenomas, and papillary neoplasms that are ≥1.0 cm): clinicopathologic and immunohistochemical analysis of 123 cases. Am J Surg Pathol.

[19] Logrado A, Constantino J, Daniel C, Pereira J, Carvalho MT, Casimiro C (2021). Low-grade dysplastic intracholecystic papillary neoplasia: a case report. Am J Case Rep.

[20] Jung W, Kim BH (2011). Biliary intraepithelial neoplasia: a case with benign biliary stricture. Korean J Hepatol.

